# The mechanisms of action of *Plasmodium* infection against cancer

**DOI:** 10.1186/s12964-021-00748-5

**Published:** 2021-07-09

**Authors:** Xiaoping Chen, Li Qin, Wen Hu, Dickson Adah

**Affiliations:** 1 State Key Laboratory of Respiratory Disease, Center of Infection and Immunity, Guangzhou Institutes of Biomedicine and Health, Chinese Academy of Sciences, Guangzhou, 510530 China; 2 CAS-Lamvac Biotech Co., Ltd, Guangzhou, 510530 China

**Keywords:** Checkpoint blockade, *Plasmodium* infection, Cancer, Immunotherapy, Mechanism, Ecological disease, Ecological counterattack therapy

## Abstract

Our murine cancer model studies have demonstrated that *Plasmodium* infection activates the immune system that has been inhibited by cancer cells, counteracts tumor immunosuppressive microenvironment, inhibits tumor angiogenesis, inhibits tumor growth and metastasis, and prolongs the survival time of tumor-bearing mice. Based on these studies, three clinical trials of *Plasmodium* immunotherapy for advanced cancers have been approved and are ongoing in China. After comparing the mechanisms of action of *Plasmodium* immunotherapy with those of immune checkpoint blockade therapy, we propose the notion that cancer is an ecological disease and that *Plasmodium* immunotherapy is a systemic ecological counterattack therapy for this ecological disease, with limited side effects and without danger to public health based on the use of artesunate and other measures. Recent reports of tolerance to treatment and limitations in majority of patients associated with the use of checkpoint blockers further support this notion. We advocate further studies on the mechanisms of action of *Plasmodium* infection against cancer and investigations on *Plasmodium*-based combination therapy in the coming future.

**Video Abstract**

**Supplementary Information:**

The online version contains supplementary material available at 10.1186/s12964-021-00748-5.

## Background

Cancer immunotherapy has been recognized as a leading breakthrough since it was selected by the journal of Science as the breakthrough of the year in 2013, especially the adoptive chimeric antigen receptor T (CAR-T) cell therapy and the immune checkpoint blockade therapy [1]. Even though CAR-T cell therapy is very effective in B cell leukemia and lymphoma, its efficacy in the treatment of malignant solid tumors is limited perhaps due to tumor immunosuppressive microenvironment and other factors [2–4]. Immune checkpoint blockade therapy, for example, the use of PD-1 antibody has been confirmed to be effective in the treatment of various advanced solid tumors including melanoma, non-small cell lung cancer, renal cancer, and so on. Immune checkpoints, such as CTLA-4 and PD-1, can be viewed as the brakes of immune cells, and the evolutional outcomes of the immune systems in animals and humans to avoid over responses that lead to immunopathogenesis. In patients with advanced tumors, cancer cells release various signal molecules that upregulate the expression of checkpoint molecules on immune cells to inhibit their function. Thus, these immune cells become inactivated “sleeping” cells that are unable to recognize and attack cancer cells. But these sleeping immune cells can be wakened and reactivated by checkpoint inhibitors [5–8]. Due to the particular importance of these work, the discoverer of anticancer activity of CTLA-4 blockage through its antibody [9], James P. Alison and the discoverer of PD-1 molecule [10], Tasuku Honjo were awarded the 2018 Nobel Prize in Physiology or Medicine.

T cells in mammals and humans are the main force against cancer, and are equipped with two set of machinery, one is co-stimulatory molecules, which can be viewed as the accelerators, and another is co-inhibitory molecules (checkpoints), which can be referred to as immune brakes. The major difference between the T cells from healthy persons and that from patients with cancer, especially those with advanced cancer, is that the latter express more checkpoint molecules, such as PD-1 through the signaling of cancer cells, while the cancer cells express the inhibitory ligands (such as PD-L1) of checkpoints. When activated T cells expressing high levels of checkpoints contacts with cancer cells with high levels of inhibitory ligands, they immediately stop their action through inhibitory signaling, thus the cancer cells escape the attack of immune cells. It has been confirmed that checkpoint blockade can recover the anticancer immune responses of these T cells [5–8]. Nevertheless, the tumor microenvironment (TME) is very complicated [11–13] even though it could be simply characterized into two categories: cold (non T cell inflamed) or hot (T cell inflamed), which is largely attributed to the levels of proinflammatory cytokine production and T cell infiltration. Those hot tumors are characterized by T cell infiltration and molecular signatures of immune activation, whereas cold tumors show significant features of T cell absence or exclusion. In general, the hot tumors present higher response rates to checkpoint inhibitors, while cold tumors (such as glioblastomas) present low mutation load and rare infiltrating immune effector cells, and are thus largely resistant to multiple immune checkpoint blockade therapies [14–17]. Besides expressing more checkpoint molecules on effector T cells, TME is infiltrated with various immunosuppressive cells, such as myeloid-derived suppressor cells (MDSCs), regulatory T cells (Tregs), tumor-associated macrophages (TAMs) and cancer-associated fibroblasts (CAFs) as well as their effector molecules, such as IL-10 and TGF-β, to form a strong immune suppressive network or TME [11–13, 18] within solid tumors. Therefore, checkpoint inhibitors alone could not systemically counteract this immunosuppressive network or microenvironment.

## *Plasmodium* immunotherapy

**Fig. 1:**
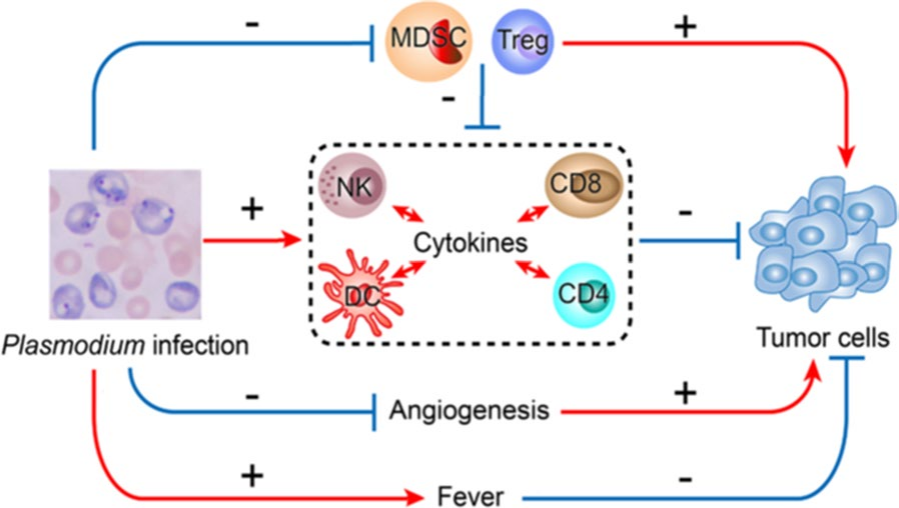
Simplified schematic diagram of *Plasmodium* infection against cancer (“ + ” or red line represents promotion, “ − ” or blue line represents inhibition). *Plasmodium* infection activates innate immune cells including natural killer (NK) cells and dendritic cells (DC) that could kill some cancer cells. These dead cancer cells would release tumor antigens that could activate acquired tumor-specific CD4^+^ and CD8^+^ T cell immunities that could more effectively kill cancer cells. *Plasmodium* infection reduces the number of immune suppressor cells including myeloid-derived suppressor cells (MDSCs) and regulatory T cells (Tregs) within tumor and inhibits their function. *Plasmodium* infection also inhibits tumor angiogenesis through multiple mechanisms. *Plasmodium* infection induces regular fever that may kill some cancer cells in cancer patients

A strategy to switch cold tumors to hot tumors is to induce a systemic Th1/proinflammatory cytokine response. Through a series of murine solid tumor model studies, we have demonstrated that *Plasmodium* infection induces Th1/proinflammatory cytokine production (including IFN-γ and TNF-α), activates innate immune cells including NK cells and dendritic cells (DCs). Activation of these innate immune cells could kill some of the cancer cells that would release tumor antigens, which then activate tumor antigen-specific T cell responses systemically in peripheral blood, spleen, tumor-draining lymph nodes and within tumor tissues, promotes NK cell and T cell infiltration and cytokine secretion (such as granzyme B) that could kill cancer cells [19]. *Plasmodium* infection simultaneously upregulates the expression levels of co-stimulatory (such as CD40L, OX-40, GITR) and co-inhibitory checkpoint molecules (such as PD-1, CTLA-4, TIM-3) on CD8^+^ T cells in tumor-bearing mice, but these CD8^+^ T cells express high levels of effector molecules (such as perforin and granzyme B) at the same time, representing the increase of their cytotoxicity. This is particularly important, because the immune balance between co-stimulatory and co-inhibitory signaling can avoid excessive immunopathogenesis and prevent the animals from dying of infection [20]. *Plasmodium* infection significantly inhibits cancer cell expression and release of MDSCs/Tregs-recruiting cytokines and chemokines, significantly reduces the numbers of MDSCs, Tregs [21], TAMs [22] and CAFs (unpublished data), and inhibits their activities in tumor tissues. *Plasmodium* infection also inhibits tumor angiogenesis through micro-RNA (miRNA) 16/322/497/17 within exosomes [23], and long noncoding RNA (lncRNA F66) [24], both of which target VEGF/VEGFR2 pathway via different mechanisms of action or through changing the functional phenotype of TAMs via engulfing malaria pigment hemozoin that blocks IGF-1/MMP9 signal pathways [22]. In addition, *Plasmodium* infection induces regular fever in humans that may also have some effect on cancer cells. We simply summarize the mechanisms of *Plasmodium* infection in the fight against cancer as shown in Fig. 1. Furthermore, our study demonstrates that *Plasmodium* parasites can serve as a vaccine vector to construct therapeutic cancer vaccine that could induce strong tumor antigen-specific T cell responses, and more effectively treat cancer in murine liver cancer model [25]. Besides the murine model studies, our global epidemiological data analysis indicates that worldwide malaria incidence is inversely associated with age-standardized all-cause cancer mortality, and with some of the malignant solid tumor (such as lung, breast, stomach and colon cancer) mortalities [26]. Based on these studies, three single-arm phase 1–2 clinical trials of *Plasmodium* immunotherapy for advanced lung cancer NCT02786589), advanced breast and liver cancers (NCT03474822), and advanced cancers (NCT03375983) have been approved and are ongoing in China.

Despite the numerous beneficial effects of *Plasmodium* infection on cancers, the clinical safety of *Plasmodium* immunotherapy might be of valid concern to some people. In order to reduce and even prevent the occurrence of the side effects from the use of *Plasmodium* for cancer immunotherapy, several measures were taken as follows. First, we use a benign form of human malaria parasite, *Plasmodium vivax* (*P. vivax*). Even though there are chloroquine-resistant *P. vivax* in some countries and areas [27–29], the *P. vivax* strain that we selected is sensitive to all antimalarial drugs, such as artemisinin and its analogs (including artesunate), quinine, chloroquine, primaquine and so on. The parasitemia is very easy to control while using one of these antimalarial drugs. Secondly, we use the blood stage of *P. vivax* thereby avoiding the liver stage parasites that causes relapse. In comparison with bacteria and viruses, *Plasmodium* parasites have an advantage in measuring their number, for example, they only infect red blood cells, a droplet of blood for making a slide for calculating the infection rate of red blood cells (parasite density) is sufficient for a routine test per day. Our clinical research proposal requires the parasite density under 0.05%; a single low dose of artesunate would be given to the patient if his or her parasite density is reaching or over 0.05%. This is a very effective way to control the parasitemia to a safe level. The rationale of controlling parasite density under 0.05% in our clinical trials is that the low density parasitemia is able to activate immune cells such as NK cells and T cells in cancer patients (unpublished data) just as that of high density does in murine cancer models. Someone may raise concerns of the development of drug resistance upon using artesunate in such a way. But our observation and available data confirms that up to date, there are no any signs to show drug resistance of the parasites in the treatment of over 100 cancer patients, and no death due to *Plasmodium* infection among these patients, therefore the clinical safety of *Plasmodium* immunotherapy for advanced cancer is guaranteed (unpublished data).

Furthermore, to address possible concerns of public health safety while using *Plasmodium* immunotherapy, we have four lines of defense. The first line is the ecological situation of malaria vectors. Malaria is transmitted by mosquitoes of the Anopheles genus. Only four species have been considered as predominant malaria vectors throughout mainland China since the beginning of the twenty-first century, that is, Anopheles sinensis, Anopheles anthropophagus (lesteri), Anopheles minimus and Anopheles dirus. After several years of effort for malaria control and elimination, the area of distribution of the principal malaria vectors was reduced, in particular for Anopheles anthropophagus and Anopheles dirus, which nearly disappeared from their former endemic regions. Anopheles sinensis is becoming the predominant species in China, especially in southwestern areas [30–32]. The breeding ground of *Anopheles sinensis* is preferably rice fields [32], but the urban areas lack rice fields. Our clinical trial centers are located in Guangzhou, the capital city of Guangdong Province, and Kunming, the capital city of Yunnan Province, both are located in south/southwestern parts of the country. Such a location selection has profoundly reduced the possibility of malaria transmission. This can be corroborated by the fact that hundreds of thousand patients with neurosyphilis received the treatment of malaria therapy [33–35] without any documented malaria transmissions throughout the period of 1917–1960s. The second line of defense is the process of professional environmental evaluation for our collaborative hospitals, a surveillance of the environment within hospital and around for Anopheles genus during the peak season of mosquito activity was carried out, and only those hospitals without any Anopheles mosquitos were qualified for the clinical trials. This process of environmental evaluation guarantees the public health safety of *Plasmodium* immunotherapy. The third line of defense is controlling parasitemia based on our clinical trial proposal. Our collaborators use low dose of artesunate to control *Plasmodium* infected red blood cells (iRBCs) below 0.05%. In addition, artesunate is superior to chloroquine in inhibiting the development of gametocytes that can infect mosquitos [36], and even better than primaquine (a gametocyte-killing drug) in the prevention of mosquito infection [37]. Our collaborators checked the blood every day and did not find any gametocytes in our treated patients, therefore there was no need to use primaquine to kill gametocytes that is required if gametocytes are found in the peripheral blood based on the proposal. The fourth line of defense is a strict standard for patient discharge. When finishing the course of *Plasmodium* immunotherapy, patients will receive an effective combination antimalarial drug therapy to terminate the parasitemia and cure the infection. The discharge standard for patient is *Plasmodium* DNA negative tested by a high sensitive PCR. Up to date, no patient carries malarial parasites at discharge. The comprehensive applications of the four lines of defense guarantees the public health safety of *Plasmodium* immunotherapy.

## Mechanistic comparison of *Plasmodium* immunotherapy with checkpoint blockade therapy

**Fig. 2:**
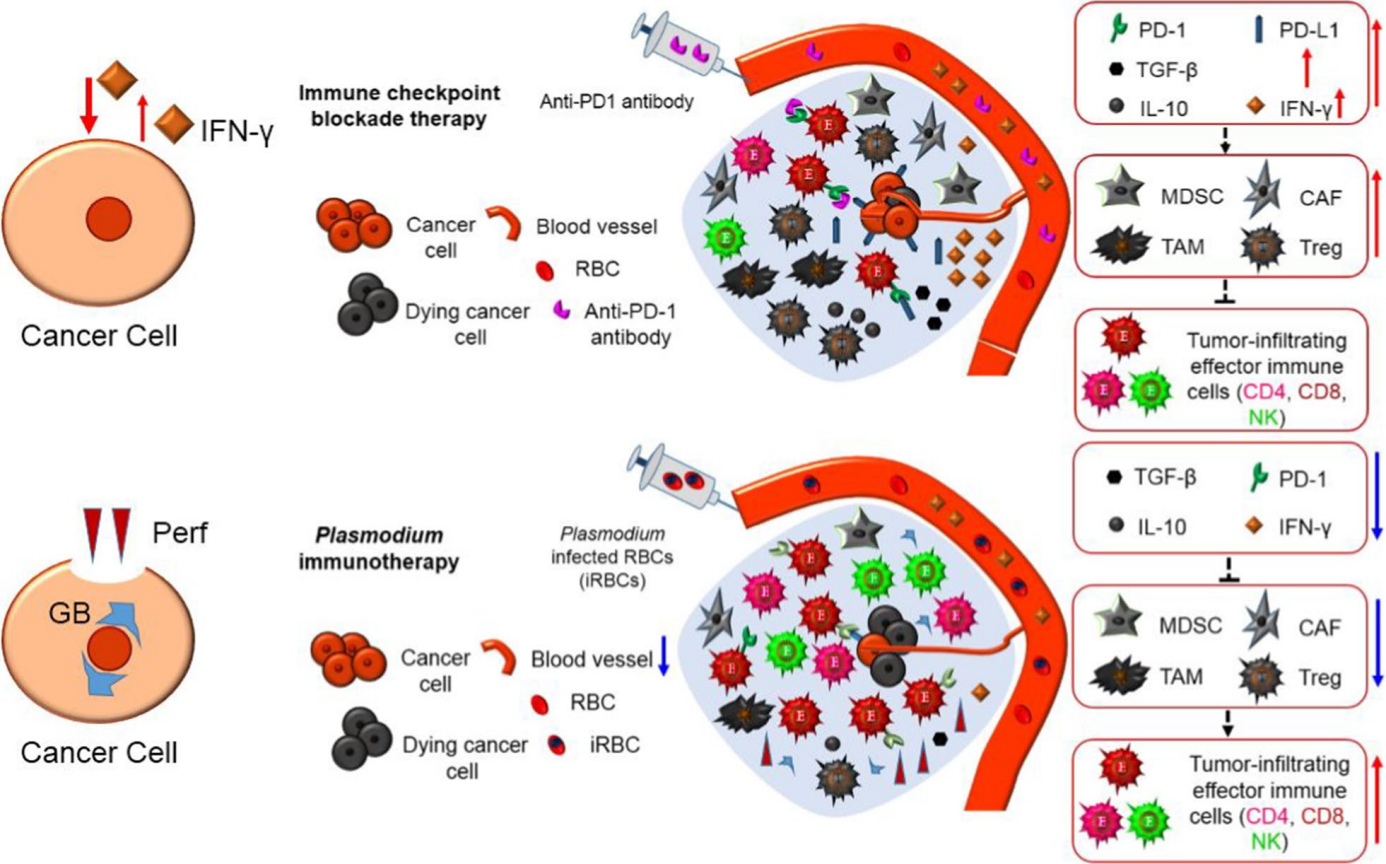
Mechanistic comparison of *Plasmodium* immunotherapy (down panel) with checkpoint blockade therapy (up panel). In some cancer patients, checkpoint blockade therapy are effective at the beginning, but are ineffective thereafter, this situation is called acquired drug resistance that is induced by IFN-γ secreted from reactivated CD8^+^ T cells. IFN-γ is a double-edged sword, it can kill cancer cells, but those that are not killed would express more PD-L1 due to the stimulation of IFN-γ, and the reactivated CD8^+^ T cells also express more PD-1 due to the irritation of this cytokine. Furthermore, IFN-γ is required for the development of immune suppressor cells, and for their immunosuppressive activities. Therefore, IFN-γ secreted by CD8^+^ T cells would increase the number of MDSCs, Tregs, TAMs and CAFs within tumor. With the recruitment of immune suppressor cells, their immunosuppressive molecules such as IL-10 and TGF-β would also increase, which in turn induce more immune suppressor cells within tumor, finally forming a vicious circle that cause the acquired drug resistance. *Plasmodium* immunotherapy activates the whole immune system from the beginning of innate immunity, counteract tumor immunosuppressive microenvironment, reduces the number of MDSCs, Tregs and other immunosuppressive cells, reduces the levels of their effector molecules such as TGF-β and IL-10, promotes the infiltration of immune cells into tumor, thus turn cold tumor into hot tumor, and inhibits tumor angiogenesis. *Plasmodium* immunotherapy stimulates CD8^+^ T cells to secrete perforin and granzyme B, but not secretes IFN-γ within tumor, thereby not inducing a vicious circle

Besides overcoming primary treatment resistance, *Plasmodium* immunotherapy can also conquer the acquired resistance. In some cancer patients, immune checkpoint blockade therapy are effective at the beginning, but are ineffective thereafter, this situation is called acquired drug resistance. The cause of this phenomenon is not due to disappearance of CD8^+^ T cell within tumor, but due to the effector molecule IFN-γ secreted from these immune cells when checkpoint inhibitor (such as PD-1 antibody) binds with the checkpoint molecule (such as PD-1). IFN-γ is a double-edged sword, it can kill cancer cells, but those that are not killed would express more PD-L1 due to the stimulation of IFN-γ, and the CD8^+^ T cells also express more PD-1 due to the irritation of this cytokine. Furthermore, IFN-γ is required for the development of MDSCs, Tregs and other immune suppressor cells, and for their immunosuppressive activities. Therefore, IFN-γ secreted by CD8^+^ T cells would increase the number of MDSCs, Tregs and other immune suppressor cells within tumor, while it kill some cancer cells. With the recruitment of immune suppressor cells, immunosuppressive molecules such as IL-10 and TGF-β secreted from these cells would also increase, which in turn induce more immune suppressor cells within tumor, finally forming a vicious circle that cause the acquired drug resistance [38–41]. This mechanism is demonstrated in Fig. 2 (up panel). Nevertheless, *Plasmodium* immunotherapy is different, it would activate the whole immune system from the beginning of innate immunity, counteract tumor immunosuppressive microenvironment, promotes the infiltration of immune cells into tumor, thus turn cold tumor into hot tumor, and inhibits tumor angiogenesis. A significant difference between *Plasmodium* immunotherapy and checkpoint blockade therapy is that, the former promotes CD8^+^ T cells to express and secrete perforin and granzyme B, but not express and secrete IFN-γ within tumor [21], even though a lot of IFN-γ are produced in peripheral blood and spleen of *Plasmodium* infected tumor-bearing mice [19], therefore reduces the level of IFN-γ within tumor [21]. Such a mechanism does not induce a vicious circle. Furthermore, *Plasmodium* immunotherapy inhibits tumor secretion of cytokines and chemokines that could recruit MDSCs, Tregs and other immune suppressor cells into tumor, therefore reduces the numbers of these cells, and decreases the level of their effector molecules (such as IL-10 and TGF-β). That is to say, *Plasmodium* immunotherapy systemically counteracts tumor immunosuppressive microenvironment, but checkpoint blockade therapy only solve a hurdle of a workstation on T cells. In addition, regarding the workstation, *Plasmodium* immunotherapy also does a similar job, downregulates the expression of PD-1 on effector CD8^+^ T cells [21] (removes brakes from a car, not just block the brakes). That is to say, *Plasmodium* immunotherapy more completely solves the hurdles (such as PD-1) on CD8^+^ T cells than does the checkpoint blockade therapy at this point. This mechanism of *Plasmodium* immunotherapy can be further explained by the down panel of Fig. 2. Because *Plasmodium* infection can systemically counteract tumor immunosuppressive microenvironment, promote infiltration of immune cells into tumor, change a cold tumor to a hot tumor, a combination of *Plasmodium* immunotherapy and checkpoint blockade therapy or a sequential treatment of both would be expected for cold tumor (such as glioblastoma, primary drug resistance to checkpoint inhibitors) or for reversion of acquired drug resistance to checkpoint inhibitors in the future studies.

## Systemic ecological counterattack therapy

Why does *Plasmodium* parasites employ diverse mechanisms to inhibit and kill cancer cells, but checkpoint inhibitors such as PD-1 antibody only solve a hurdle of workstation on T cells? The answer is that checkpoint inhibitors are artificially designed based on checkpoint molecules, and *Plasmodium* parasites are naturally existent without any artificial design. This needs to be described from the nature of cancer. From the viewpoint of immunology, cancer is a result of long term interaction and struggle against each other between cancer cells and the immune system, and is formed through a process called immunoediting. At the early stage of tumorigenesis and development, the immune system dominates the interaction, and can clear most part or even all of cancer cells, and this stage is called “Elimination”. Over time, some cancer cells develop resistance to the killing of the immune system, these cancer cells can therefore coexist with the immune system in the body. At this stage, the immune system cannot clear all of the cancer cells, even though cancer cells also cannot multiply and progress unhindered. Thus cancer cells reproduce and are cleared in a balanced state, this is called “Equilibrium”. This stage can be viewed as the dormancy stage of cancer. When most cancer cells acquire resistance to the killing of the immune system, they can reproduce fast. This stage is called “Escape”. The above notion is called immunoediting theory or three Es theory [42–45]. Most of the cancer patients in clinics are at the stage of escape. The immunological characteristic of this stage is that cancer cells secrete a series of signal molecules that allow the immune system to sleep, disabling anticancer activities. Malignant solid tumor also secretes signal molecules to recruit immature immune cells and other cells to enter the tumor, then educate, or reprogram them to become immune suppressor cells (such as MDSC, Treg, TAM and CAF) that could secrete immunosuppressive molecules (such as IL-10 and TGF-β), therefore forming the immunosuppressive microenvironment [11–13, 18]. All of these are intrinsically based on the fact that cancer cells can secrete a series of signal molecules that could inhibit the immune system. A living *Plasmodium* parasite is a very strong antigen carrier that could sensitize and activate the whole immune system that has been suppressed by tumor. For example, *Plasmodium* infection activates innate immune cells through their pathogen-associated molecular patterns (PAMPs) that could interact with the pattern recognition receptors (PRRs) of the immune cells [46–48], reprograms the cancer cells, changes their gene expression profiles through the action of exosomes secreted from the parasite-infected red blood cells and activated immune cells [21, 23], restricts them to secrete MDSCs/Tregs-recruiting and immunosuppressive molecules [21], and inhibits tumor angiogenesis through a series of mechanisms [22–24]. All of these are based on the ongoing activation of the whole immune system due to a sustainable infection of *Plasmodium* parasites. In principle, almost all pathogen infections possess similar characteristics, including the infections with viruses and bacteria. But infection with virus could be solved very fast by the immune system, such as influenza virus [49] or adenovirus [50]. Otherwise, the outcome of infection could be very severe, such as smallpox virus [51] or *yersinia pestis* [52]. Furthermore, some pathogen infection could lead to disability, such as the infection with poliovirus [53]. Of course, these pathogens could be attenuated through gene modification, but the attenuation of a pathogen would lead to a very short duration of infection, the attenuated pathogen will be cleared very fast by the immune system, unable to induce sustainable activation of the immune system, similar to the infection with a wild-type adenovirus. The root cause of these phenomena is that, these pathogens are prokaryotic cells (bacteria), or even without cellular structures (viruses), unable to evolve a specialized antigenic variation system for coping with the immune system of the hosts, and the pathogens would be cleared very quickly when the infected hosts develop acquired immunity (about one to two weeks). Thus, these pathogens are unable to induce a durable or a second infection. Therefore these pathogens can only be used for one time (cannot be used for the second time) [54–57]. But *Plasmodium* parasites are different, because they are eukaryotic protozoa that have nucleus and chromosomes in which there is a histone machinery that fine-tunes the regulation of gene expression, such that they have developed a specialized antigenic variation system (such as *var* gene family of *P. falciparum*) [58–60] to cope with the immune system through a long term coevolution with their hosts. *Plasmodium* parasites have the ability to escape the clearance of the immune system through diverse antigenic variation mechanisms that induce a sufficient and durable immune response to achieve an anticancer efficacy, even though a duration (4–8 weeks) of acute to subacute infection (not necessary to use chronic infection) is enough as a treatment course (unpublished data). It is worth noting that chronic malaria could induce the anergy of Plasmodium antigen-specific CD8^+^ T cells [61], but not the anergy of other antigen-specific (such as tumor antigen-specific) CD8^+^ T cells. For example, unlike HIV infection, chronic malaria does not provoke the occurrence of opportunistic infection in humans. Furthermore, they can repeatedly infect their hosts [62, 63], which necessitates multiple courses of treatment, and can easily be controlled by artemisinin, so that the safety of *Plasmodium* immunotherapy can be guaranteed.

Since 1970s, cancer genetics and cancer biology have been viewed as the mainstream of cancer research. The notion that “cancer is a disease due to genetic mistake of normal cells” was leading this field. On the clear-cut contrast, the viewpoint that “cancer is a systemic disease due to imbalance of the immune system” was payed much less attention. In the past decades, there have been significant advances in immunology (especially cancer immunology and immuno-oncology), a historically important consensus among cancer researchers is emerging about the causality of chronic inflammation and altered immunity in driving malignant development and progression [64–66]. Research scientists in this field have evolved from only keeping watchful eyes on cancer cells in the past to paying close attention to tumor stroma microenvironment, inflammation and changes of the immune system that influence tumor origin, dormancy and development, leading to the diversification of cancer treatment, namely the coming of the era of cancer immunotherapy. That is to say that in the past, oncology is only focused on “seed” (cancer cells), but nowadays oncologists simultaneously pay attention to “seed, soil, water and environment” (tumor stroma and microenvironment), a development of the hypothesis of “seed and soil” earlier proposed by the English surgeon, Stephen Paget in 1889 for explaining the mechanisms of tumor metastasis [67–69]. Thus, cancer biology develops from a cell biology to an ecology, the concept of evolution has come into the basic research of oncology and the field of cancer treatment. Cancer cells originate from host cells, with very similar characteristics of the host cells, leading to the difficulty of cancer treatment. Since cancer cells possess heterogeneity and plasticity, all treatment with drugs become selective pressures. While killing drug sensitive cancer cells, those insensitive cancer cells will be selected to become a dominant population. Facing such a natural selection, effective cancer treatment should be similar to that of other highly gene-mutated diseases (such as HIV infection), a drug combination (such as the combination of different targeted drugs) should be needed. But the genome of cancer cell is much large than that of HIV, therefore its space of mutation is also bigger than that of this virus. Even in the future when a combination with multiple drugs or multiple treatments becomes advanced, complete clearance of cancer cells will still be a great challenge, since cancer is an ecological disease involving seed, soil, water and environment, any treatment that merely target the seed (cancer cells) is bound to fail.

**Fig. 3:**
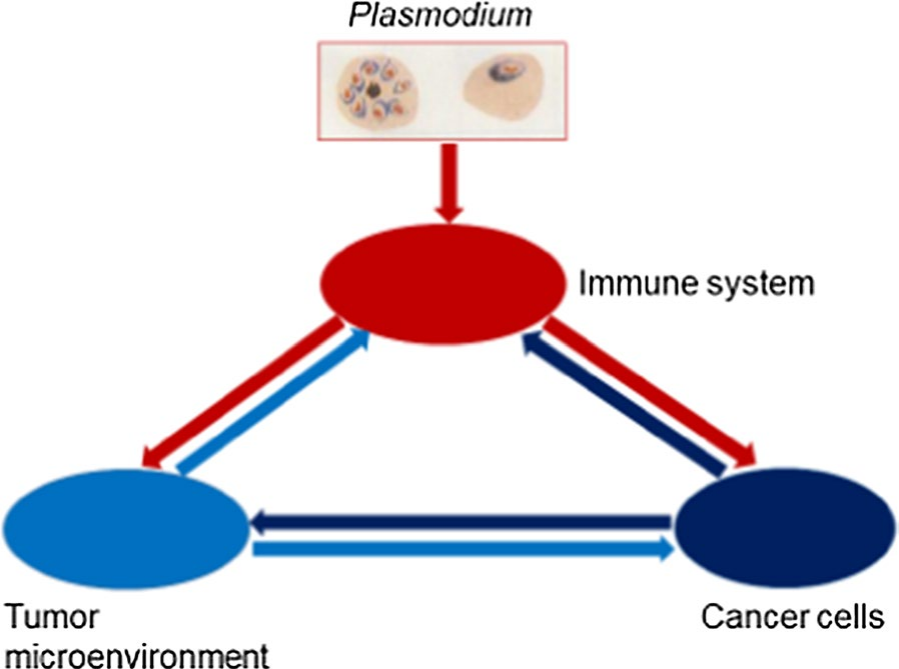
Schematic diagram of the notion that cancer is an ecological disease and that *Plasmodium* immunotherapy is a systemic ecological counterattack therapy (or ecological therapy) for this ecological disease. Cancer cells secrete a series of immunosuppressive molecules to reprogram immune cells and make a counterespionage to the whole immune system, by recruiting, educating and training the tumor stroma cells to construct an immunosuppressive tumor microenvironment, which in turn secretes a series of molecules that promote tumor growth and metastasis, and further suppress the immune system. *Plasmodium* immunotherapy comprehensively activates the immune system that has been inhibited by cancer cells, promotes immune cells to secrete a series of signal molecules (some of these molecules exist within secreted exosomes) that reprogram cancer cells, change their gene expression profiles, inhibit their ability to secrete a series of signal molecules, thereby remodeling the tumor microenvironment by turning the immune-suppressive milieu to immune-supportive milieu

Here, we propose the notion that cancer is an ecological disease. The fundamental connotation of this notion is that, during a long term interaction with the immune system, cancer cells experience immunoediting, from elimination, equilibrium to escape, during which the tumor establishes an immunosuppressive microenvironment, realize a fully ecological control of immune cells including those within tumor microenvironment, in remote sites, and even the whole immune system through secretion of exosomes that contain immunosuppressive molecules [70–73]. That is to say, cancer cells construct the ecological environment that help them to grow and metastasize, reprogram immune cells, realize a full-scale counterespionage and control of the immune system. In such a circumstance, all kinds of therapies at present may be insufficient in the fight against a deranged ecological system. Therefore, all current target-based drugs (including cancer cell-based targeted drugs and immune cell-based checkpoint inhibitors) may be unable to provide a full-scale ecological counterattack therapy, because all these drugs are single target-based, even if some multiple target-based drugs are developed in the future, their targets will still be limited. Interestingly, *Plasmodium* immunotherapy accurately aligns with the philosophy of a full-scale ecological counterattack therapy (or ecological therapy, Fig. 3). *Plasmodium* immunotherapy activates the “sleeping” immune system that has been suppressed by cancer cells. Furthermore, *Plasmodium* infected red blood cells and the activated immune cells secrete exosomes to act on cancer cells at any sites of the body. These exosomes reprogram cancer cells by changing their gene expression profiles. *Plasmodium* therapy also inhibits the expression of signaling molecules that recruit immunosuppressive cells (such as MDSCs, Tregs, TAMs, and so on) into tumor tissues, and inhibit the function of these immunosuppressive cells (for example, the secretion of IL-10 and TGF-β), thereby systemically counteracting the tumor immunosuppressive microenvironment. Our previous studies also demonstrated that *Plasmodium* infection inhibits tumor angiogenesis, cutting off its nutrition supply. Our murine cancer model studies have demonstrated that *Plasmodium* immunotherapy significantly promotes T cell infiltration into tumor tissue, downregulates PD-1 expression on T cells, upregulates perforin and granzyme B expression within T cells, and enhances tumor-specific cellular immune responses within tumors. In addition, *Plasmodium* infection significantly inhibit epithelium-mesenchymal transition (EMT), therefore prevent metastasis and relapse [74], and may also overcome drug resistance of tumor. On the basis of above mentioned mechanisms, we consider that *Plasmodium* immunotherapy may be a broad-spectrum anticancer treatment.

## Conclusions

After comparing the mechanisms of action of *Plasmodium* immunotherapy with those of immune checkpoint blockade therapy, we propose the notion that cancer is an ecological disease and that *Plasmodium* immunotherapy is a systemic ecological counterattack therapy (or ecological therapy) for this ecological disease. Checkpoint blockade is a single targeted immunotherapy that only solves a hurdle of workstation on T cells, may be ineffective for cold tumor, and may induce acquired drug resistance of hot tumor through a vicious circle due to the effect of IFN-γ secreted from reactivated CD8^+^ T cells. *Plasmodium* infection activates the whole immune system that has been suppressed by tumor. This multi-targeted mechanisms of inhibition by *Plasmodium* makes it a suitable candidate for cancer immunotherapy to overcome the weakness of single targeted therapies. We advocate further studies on the mechanisms of action of *Plasmodium* infection against cancer and investigations on *Plasmodium*-based combination therapy in the coming future.

## Data Availability

Not applicable.

## Abbreviations

CAR-T: Chimeric antigen receptor T cell
PD-1: Programmed death-1
PD-L1: Programmed death ligand 1
TME: Tumor microenvironment
MDSCs: Myeloid-derived suppressor cells
Tregs: Regulatory T cells
TAMs: Tumor-associated macrophages
CAFs: Cancer-associated fibroblasts
IL-10: Interleukin 10
TGF-β: Transforming growth factor β
Th1: T helper cell type 1
IFN-γ: Interferon γ
TNF-α: Tumor necrosis factor α
NK: Natural killer
DCs: Dendritic cells
lnc RNA: Long noncoding ribonucleic acid
VEGF: Vascular endothelial growth factor
VEGFR2: Vascular endothelial growth factor receptor 2
IGF-1: Insulin-like growth factor 1
MMP9: Matrix metalloproteinase 9
iRBCs: Infected red blood cells
PCR: Polymerase chain reaction
PAMPs: Pathogen-associated molecular patterns
PRRs: Pattern recognition receptors
HIV: Human immunodeficiency virus
